# Seroprevalence of *Toxoplasma gondii* infection in zoo and domestic animals in Jiangxi Province, China

**DOI:** 10.1051/parasite/2017007

**Published:** 2017-02-22

**Authors:** Houqiang Luo, Kun Li, Hui Zhang, Ping Gan, Muhammad Shahzad, Xiaoxing Wu, Yanfang Lan, Jiaxiang Wang

**Affiliations:** 1 College of Animal Sciences, Wenzhou Vocational College of Science and Technology Wenzhou 325006 PR China; 2 College of Veterinary Medicine, Huazhong Agricultural University Wuhan 430070 PR China; 3 Jiangxi Animal Disease Prevention and Control Center Nanchang Jiangxi 330096 PR China; 4 University College of Veterinary and Animal Sciences, The Islamia University of Bahawalpur 63100 Pakistan; 5 College of Animal Science, Yangtze University Jingzhou 434023 PR China

**Keywords:** *Toxoplasma gondii*, Zoo animals, Domestic animals, Prevalence

## Abstract

*Toxoplasma gondii* is a zoonotic protozoan parasite that infects a wide range of warm-blooded animals throughout the world. In the present study, antibodies to *T. gondii* were determined using a commercial indirect hemagglutination (IHA) test in wild animals in a zoo. Three of 11 giraffes (*Giraffa camelopardalis*) (27%), 1 of 5 wolves (*Canis lupus laniger*) (20%), 1 of 6 hippopotamuses *(Hippopotamus amphibious)* (17%), and 2 of 9 tundra swans (*Cygnus columbianus*) (22%) were found to be positive. No antibodies were detected in leopards (*Panthera pardus*), wild geese (*Anser cygnoides*), and Eastern grey kangaroos (*Macropus giganteus*)*.* Domestic species from 13 counties of Jiangxi Province, China were also investigated by an indirect hemagglutination (IHA) test. Thirty-five of 340 goats (10%), 94 of 560 water buffaloes (17%), and 4 of 35 cattle (11%) were found to be seropositive. This is the first report of *T. gondii* infection in animals kept in zoos and domestic animals in this province.

## Abstract (fr)

*Toxoplasma gondii* est un protozoaire parasite zoonotique qui infecte une large gamme d’animaux à sang chaud à travers le monde. Dans la présente étude, les anticorps dirigés contre *T. gondii* ont été déterminés en utilisant un test commercial d’hémagglutination indirecte (IHA) chez des animaux sauvages gardés dans un zoo. Trois (27 %) parmi 11 girafes (*Giraffa camelopardalis*), 1 (20 %) parmi 5 loups (*Canis lupus laniger*), 1 (17 %) parmi 6 hippopotames (*Hippopotamus amphibious*) et 2 (22 %) parmi 9 cygnes siffleurs (*Cygnus columbianus*) ont été trouvés positifs. Aucun anticorps n’a été détecté chez des léopards (*Panthera pardus*), des oies sauvages (*Anser cygnoides*) et des kangourous (*Macropus giganteus*). Les espèces domestiques provenant de 13 comtés de la province de Jiangxi, en Chine, ont également fait l’objet d’une enquête par hémagglutination indirecte. Trente-cinq chèvres parmi 340 (10 %), 94 buffles d’eau parmi 560 (17 %) et 4 bovins parmi 35 (11 %) ont été retrouvés séropositifs. Ceci est le premier rapport sur l’infection par *T. gondii* chez les animaux gardés dans des zoos et les animaux domestiques de cette province.

## Introduction

*Toxoplasma gondii* is an intracellular protozoan parasite that infects a broad range of warm-blooded animals worldwide [4]. Despite the wide distribution of *T. gondii*, limited information is available on this parasite in wild and domestic animals in China [1]. A study was therefore designed to determine the seroprevalence of *T. gondii* in zoos and domestic animals in Jiangxi province, China.

Jiangxi province is located in the southeast of China (northern latitudes of 24°29′–30°04′ and eastern longitudes of 113°34′–118°28′). This province has an abundance of domestic and wild animals given its subtropical, humid monsoon climate, with average annual precipitation of 1341–1940 mm.

## Materials and methods

### Serum samples

Blood samples were collected from 39 zoo animals and randomly selected domestic animals from different farms (Tables 1 and 2). Blood samples were collected from the caudal vein by local veterinary practitioners. After collection, each of the blood samples was centrifuged at 1000 × *g* for 10 min, and serum was separated and stored at −20 °C until further analysis.

**Table 1.** Prevalence of *Toxoplasma gondii* infection in zoo animals determined by indirect hemagglutination in Jiangxi province, China in 2016.

Species	No. tested	No. positive	Prevalence %
Giraffe (*Giraffe camelopardalis*)	11	3	27.3
Wolf (*Canis lupus laniger*)	5	1	20.0
Hippopotamus (*Hippopotamus amphibious*)	6	1	16.7
Swan (*Cygnus columbianus*)	9	2	22.2
Leopard (*Panthera pardus*)	3	0	0
Wild goose (*Anser cygnoides*)	2	0	0
Kangaroo (*Macropus giganteus*)	3	0	0
Total	39	7	17.9

**Table 2.** Prevalence of *Toxoplasma gondii* infection in goats, water buffaloes and domestic cattle, determined by indirect hemagglutination (IHA) test in Jiangxi province, China in 2016.

	Goats	Water buffaloes^a^	Cattle
County	Prevalence % (No. positive/No. tested)	Prevalence % (No. positive/No. tested)	Prevalence % (No. positive/No. tested)
Pingxiang	5.5 (3/55)	0 (0/15)	11.4 (4/35)
Ji’an	10.0 (2/20)	17.5 (21/120)	
Xinyu	20.0 (4/20)	34.3 (12/35)	
Jingdezhen	15.0 (3/20)	22.9 (8/35)	
Yichun	10.0 (2/20)	15.6 (7/45)	
Nanchang	11.4 (4/35)	26.3 (21/80)	
Shangrao	0 (0/10)	17.1 (6/35)	
Yingtan		6.7 (1/15)	
Ganzhou	0 (0/20)	5.7 (2/35)	
Jiujiang	12.1 (17/140)		
Fuzhou		8.0 (2/25)	
Gaoan		5.0 (3/60)	
Yudu		18.3 (11/60)	
Total	10.3 (35/340)	16.8 (94/560)	11.4 (4/35)

a There were significant differences in the prevalence of *T. gondii* infection in water buffaloes in different counties (*p* < 0.01; *χ*^2^ = 31.203).

### Determination of antibodies against *T. gondii*

Each of the serum samples was tested for antibodies against *T. gondii* by employing a commercial indirect hemagglutination test (IHA, Lanzhou Veterinary Research Institute of the Chinese Academy of Agricultural Sciences) according to the manufacturer’s instructions. The test was considered positive when a layer of agglutinated erythrocytes formed in wells using the serum dilutions of 1:64 or higher, and positive and negative controls were included in each test.

Statistical analysis was performed by chi-square testing with SPSS (Statistical Analysis System, Version 18.0). The differences were considered statistically significant when *p* < 0.05.

## Results and discussion

Antibodies to *T. gondii* were found in 7 out of 39 zoo animals (17.9%) (Table 1). The data for domestic animals are shown in Table 2.

The present study reported a prevalence of 12.1% for *T. gondii* infection in goats in Jiangxi province, which was in the same range as the prevalence of infection reported in goats (13.4%) in the Hubei and Hunan provinces of China [2, 4], and significantly higher than the prevalence in the northeastern part of the country (8.92%) [5].

The prevalence of *T. gondii* infection in buffaloes and cattle was 16.8% and 11.4%, respectively, which was lower than the prevalence of *T. gondii* infection observed in yaks from the same family (21.7% and 29.1% in 2012 and 2013) on the Qinghai-Tibetan plateau of China [3]. As more than three million head of cattle are farmed in this province, there is a high risk of transmission to other animals.

Previously, numerous studies were conducted on the seroprevalence of *T. gondii* in humans. However, limited information is available regarding the seroprevalence of this protozoan in wildlife, especially in zoological gardens in China, with two reports concerning the Shanghai and Beijing zoological gardens [6, 7]. High levels of *T. gondii* infection are found in felids. Since this protozoan can be shed by felids, this might have contributed to the spread of *T. gondii* in the zoo. Therefore, the personnel working in the zoo or the people visiting the zoo should be aware of the procedures required to reduce the potential zoonotic threat of *T. gondii* [7].

The prevalence of *T. gondii* infection in zoo animals (17.9%) and in domestic buffaloes (16.8%), cattle (11.4%), and goats (10.3%) in Jiangxi province indicates *T. gondii* exposure in several animal species. Currently, no information is available on clinical toxoplasmosis in animals in this province. Further studies are needed to determine the prevalence of viable *T. gondii* in cattle and buffalo tissues because these animals are considered resistant to this protozoan [1].

## Conflict of interest

None of the authors have any conflict of interest.
